# Supplementary material for: Measurement error in continuous endpoints in randomised trials: Problems and solutions
Source: Stat Med. 2019 Sep 2;38(27):5182–96. doi: 10.1002/sim.8359 (PMC6900013; doi:10.1002/sim.8359)
Supplement: Supplementary file 1 — SIM_8359‐Supp‐0001‐Supplementary_Material__Measurement_error_in_continuous_endpoints_in_ran....pdf [file SIM-38-5182-s001.pdf]

Supplementary Materials

These are the supplementary materials accompanying the paper 'Measurement error in continuous endpoints in randomised trials: problems and solutions' by L. Nab et al. The supplementary materials are structured as follows. In section 1 we discuss two more example trials for illustration of measurement errors in an endpoint. In section 2 we explain why and under which assumptions ignoring measurement error will lead to incorrect inference. Section 3 provides an explanation of corrected effect estimators (and why these are consistent) and explains the methods used for confidence interval estimation. Section 4 provides a prove that measurement error depending on prognostic factors does not introduce bias in the treatment effect estimator.

1 Illustrative examples

We introduce here two additional example trials from literature, hypothesize that these trial could also have used endpoints measured with error to illustrate how the use of an endpoint that is contaminated with error would affect trial inference. We assume that the original endpoints used in our example trials are measurement error free.

1.1 Example trial 2: energy expenditure

Poehlman and colleagues [1] studied the effects of endurance and resistance training on total daily energy expenditure in a randomised trial of young sedentary women. Participants were randomized to one of three six-month during exercise programmes: endurance training, resistance training or the control arm. Some controversy regarding the effect of exercise training on total energy expenditure (TEE) existed at the time of the start of the trial, partly because of the difficulty to assess daily energy expenditure [1]. Starting 72 hours after completion of the training program, TEE of the participants was measured by doubly labelled water during a ten day period, which is considered the gold standard in measuring energy expenditure in humans [2]. In short, the study found no evidence for an effect of resistance and endurance training (compared to placebo) on total energy expenditure. Post-trial, measured TEE was higher in the control arm than in the two intervention arms. Table 1 shows the decrease in TEE of the women exposed to the existence training programme versus the placebo arm.

1.2 Example trial 3: rheumatoid arthritis disease activity

The U-Act-Early trial tested the efficacy of a new treatment strategy for rheumatoid arthritis (RA) in patients with newly diagnosed RA [3] in a three-arm trial: tocilizumab plus methotrexate versus tocilizumab only versus methotrexate only, all as initial treatment. For endpoint assessment, this trial used a validated RA disease activity measure (the Disease Activity Score 28, DAS28) [4]) which is commonly used and recommended to measure endpoints in RA clinical trials [5, 6]. In short, the trial showed that immediate initiation of tocilizumab with or without methotrexate is more effective than methotrexate alone to achieve sustained remission in newly diagnosed RA patients. The difference in mean DAS28 score in the tocilizumab plus methotrexate versus methotrexate only group after 24 weeks is shown in Table 1. The sample size of the former groups reported in Table 1 is based on measurements available at 24 weeks of follow up.

Tab. 1: Impact of classical measurement error on Type-II error

	Effect estimate	Standard error	Sample Size	$\rho^{\ddagger}$	Type-II error*
Trial 1	$6.9^{\dagger}$	$1.27^{\dagger}$	$393^{\dagger}$	0	-
		2.43	108	0	20%
		2.71	108	1/5	29%
		2.45	132	1/5	20%
Trial 2	$-246^{\dagger}$	$369^{\dagger}$	$35^{\dagger}$	0	-
		88.7	600	0	20%
		109	600	1/3	38%
		88.7	900	1/3	20%
Trial 3	$-1.4^{\dagger}$	$0.08^{\dagger}$	$198^{\dagger}$	0	-
		0.41	8	0	18%
		0.50	8	3/7	41%
		0.44	12	3/7	18%

† Effect estimates, standard errors and sample sizes are based on results in papers by Makridis et al. [8] (trial 1), Poehlman et al. [1] (trial 2) and Bijlsma et al. [3] (trial 3).

‡ Proportion of observed variance in endpoints due to measurement error.

∗ Type-II error calculations are based on results provided in section 3.1.

A common alternative approach to measure energy expenditure (example trial 2) is by a accelerometer, that measures body movement via motion sensors to assess energy expenditure (e.g. [2]). As compared to double labelled water (example trial 2), the accelerometer is cheaper, but less accurate [2]. Lastly, instead of endpoint assessment by DAS28 (example trial 3), where assessment is done by trained medical staff [4], trials could alternatively use the patient-based RA disease activity score (PDAS), where endpoint assessment is done by the patient [7].

For the example trial in the paper and each of the aforementioned example trials here, in Table 1 we show to what extent the Type-II of a test for treatment effect changes when a hypothetical lower standard of endpoint measurement would have been used introducing classical measurement error. The table clearly shows the anticipated increase in Type-II error with increasing error at the same sample size.

2 Measurement error structures

Consider a two-arm randomized controlled trial that compares the effects of two treatments ($X \in \{0, 1\}$), where 0 may represent a placebo treatment or an active comparator. Let Y denote the true (or preferred) trial endpoint and Y^* an error prone operationalisation of Y. We will assume that both Y and Y^* are measured on a continuous scale. Throughout, we assume that Y^* is measured for all $i = 1, \dots, N$ randomly allocated patients in the trial. We assume that the effect of allocated treatment ($X \in \{0, 1\}$) on preferred endpoint Y is defined by the linear model

$$Y = \alpha_Y + \beta_Y X + \varepsilon, \tag{1}$$

where β_Y defines the treatment effect on the endpoint, and ε has expected mean 0 and variance σ^2. Throughout, we assume that X is fixed. Further, we assume that model 1 is inestimable from the

observed data because the endpoint Y^* instead of Y was measured. We will assume that the relation between Y and Y^* is given by a linear model,

$$Y^* = \theta_0 + \theta_1 Y + e, \tag{2}$$

where e is a random variable whose distribution is independent of ε, Y and X. The parameters θ_0 and θ_1 define the relation between Y and Y^*, where it is assumed that θ_1 does not equal 0. We assume that both parameters θ_0 and θ_1 are estimable only in the external calibration sample comprising individuals not included in the trial ($j = 1, \dots, K$).

Simple OLS regression estimators for β_Y, α_Y and σ^2 (the variance of the errors ε) in (1) are,

$$\begin{aligned}
\hat{\beta}_{Y^*} &= \frac{\sum_i (X_i - \bar{X})(Y_i^* - \bar{Y^*})}{\sum_i (X_i - \bar{X})^2}, && (3)\\
\hat{\alpha}_{Y^*} &= \bar{Y^*} - \hat{\beta}_{Y^*}\bar{X}, && (4)\\
\omega_i &= Y_i^* - \hat{\alpha}_{Y^*} - \hat{\beta}_{Y^*} X_i, && (5)\\
s^2 &= \frac{1}{N-2}\sum_i \omega_i^2, && (6)
\end{aligned}$$

respectively. In a two-arm trial, the interest is in making inferences about β_Y, which cannot be directly estimated because in the trial the endpoint of interest Y was replaced by Y^*. In the following we will show: a) that $\hat{\beta}_{Y^*}$ may be a poor estimator for β_Y (section 3.1-3.4), and b) how adjustments to $\hat{\beta}_{Y^*}$ using information from the calibration model described by (2) can improve inference about the treatment effect (section 4). As a starting point, in the following section relevant and known properties are defined for the special case that $Y^* = Y$, which is then followed by the properties under different measurement error structures for Y^* in subsequent sections.

2.1 No measurement error

Consider the hypothetical case that Y^* is a perfect proxy for Y, i.e. $Y^* = Y$. By using that $Y = \alpha_Y + \beta_Y X + \varepsilon$, as defined in (1), it follows that:

$$Y^* = \alpha_Y + \beta_Y X + \varepsilon.$$

From standard regression theory (e.g. [9]), we know that if the errors ε satisfy the regular Gauss-Markov assumptions [9] and their variance is defined by σ^2, the OLS estimators $\hat{\beta}_Y^*$, $\hat{\alpha}_Y^*$, and s^2 (defined by 3, 4, and 6, respectively) are Best Linear Unbiased Estimators (BLUE) for β_Y, α_Y, and σ^2, respectively.

Moreover, if the ε are independently and identically (iid) normally distributed, the OLS estimators $\hat{\beta}_{Y^*}$ and $\hat{\alpha}_{Y^*}$ (defined in 3 and 4, respectively) are the Maximum Likelihood Estimators (MLE) of β_Y and α_Y, respectively. Note that the errors ε satisfy the Gauss-Markov assumptions if we assume that they are iid normally distributed with mean 0 and constant variance σ^2.
Hypotheses for the treatment effect β_Y, can be defined by

$$\begin{aligned}
H_0 &: \quad \beta_Y = \beta_0,\\
H_A &: \quad \beta_Y \neq \beta_0.
\end{aligned}$$

Under normality of the error terms ε, the OLS estimator $\hat{\beta}_Y^*$ defined in (3) is the MLE for β_Y and s^2 is an unbiased estimator for σ^2, the following is known for the Wald test:

$$T = \frac{\hat{\beta}_{Y^*} - \beta_0}{\sqrt{\widehat{\text{Var}}(\hat{\beta}_{Y^*})}} \sim t_{N-2}, \tag{7}$$

where,

$$\widehat{\text{Var}}(\hat{\beta}_{Y^*}) = \frac{s^2}{\sum_i (X_i - \bar{X})^2}. \tag{8}$$

Assuming no measurement error in Y and X, under H_0, T follows a Student's t distribution with $N-2$ degrees of freedom [9]. Under H_A, T follows a Student's t distribution with $N-2$ degrees of freedom and non-centrality parameter $(\beta_Y - \beta_0)/\sqrt{\widehat{\text{Var}}(\hat{\beta}_{Y^*})}$.

2.2 Classical measurement error

There is classical measurement error in Y^* if Y^* is an unbiased proxy for Y [10]:

$$Y^* = Y + e, \tag{9}$$

where E$[e] = 0$ and Var$(e) = \tau^2$ and e mutually independent of Y, X, ε (in (1)).
Using that $Y = \alpha_Y + \beta_Y X + \varepsilon$ from (1), it follows that:

$$Y^* = \alpha_Y + \beta_Y X + \varepsilon + e.$$

Given the aforementioned assumptions, the sum of e and ε, $\delta_1 = e + \varepsilon$, has variance $\text{Var}(\delta_1) = \sigma^2 + \tau^2$. It follows that if the errors δ_1 satisfy the Gauss-Markov assumptions, $\hat{\beta}_{Y^*}$ in (3) remains a BLUE estimator for β_Y. Also, $\hat{\alpha}_{Y^*}$ in (4) and s^2 in (6) remain BLUE estimators for α_Y and the variance of δ_1, respectively.

Further, if δ_1 is iid normally distributed with mean 0 and variance $\sigma^2 + \tau^2$, then $\hat{\alpha}_{Y^*}$ is the MLE for α_Y and $\hat{\beta}_{Y^*}$ is the MLE for β_Y. Obviously, given that $\sigma^2 > 0$ and $\tau^2 > 0$, the variance of the OLS regression estimator $\hat{\beta}_{Y^*}$ is larger if there is classical measurement error in the outcome compared to the case when there is no measurement error. Under the null hypothesis, the Wald test-statistic T defined in (7) still follows a Student's t distribution with $N-2$ degrees of freedom. However, under the alternative hypothesis, the non-centrality parameter of T, $(\beta_Y - \beta_0)/\sqrt{\widehat{\text{Var}}(\hat{\beta}_{Y^*})}$, will be smaller in the presence of classical measurement error.

To summarize, in the presence of only classical measurement error, Type-II error for detecting any given treatment effect increases, Type-I error is unaffected and the treatment effect estimator is unbiased MLE under standard regularity conditions.

2.2.1 Heteroscedastic classical measurement error

In the preceding we assumed that the Gauss-Markov assumptions were met. But notably, in the case that the variance of the errors e in (9) varies per treatment arm, the errors are no longer homoscedastic (as needed to satisfy the Gauss-Markov assumptions) but heteroscedastic. In the case of this type of heteroscedastic classical measurement error, it can be shown that the variance of β_{Y^*} will be underestimated by the default estimator of the variance of $\hat{\beta}_{Y^*}$ defined by (8), affecting both Type-I and Type-II error.

2.3 Systematic measurement error

There is systematic measurement error in Y^*, if Y^* systematically depends on Y. Assuming this dependence is linear, the relation between Y^* and Y can be defined as:

$$Y^* = \theta_0 + \theta_1 Y + e, \tag{10}$$

where $\text{E}[e] = 0$ and $\text{Var}(e) = \tau^2$. Throughout, we assume systematic measurement error if $\theta_0 \neq 0$ or $\theta_1 \neq 1$ (and of course, $\theta_1 \neq 0$ in all cases). We assume mutual independence between e and Y, X, ε (in 1). Naturally, if $\theta_0 = 0$ and $\theta_1 = 1$ the measurement error is of the classical form.

By using that $Y = \alpha_Y + \beta_Y X + \varepsilon$ from (1), it follows that:

$$Y^* = \theta_0 + \theta_1 \alpha_Y + \theta_1 \beta_Y X + \theta_1 \varepsilon + e.$$

Given the aforementioned assumptions, $\delta_2 = \theta_1 \varepsilon + e$ with expected variance $\theta_1^2 \sigma^2 + \tau^2$. It follows that under the Gauss-Markov assumptions, $\hat{\beta}_{Y^*}$ defined in (3) is BLUE for $\theta_1 \beta_Y$, and $\hat{\alpha}_{Y^*}$ defined in (4) is BLUE for $\theta_0 + \alpha_Y$ and s^2 defined in (6) is BLUE for the variance of δ_2 (i.e. $\theta_1^2 \tau^2 + \sigma^2$). Conversely, $\hat{\beta}_{Y^*}$ is no longer BLUE for β_Y. Note that in this case s^2 is BLUE for $\theta_1^2 \sigma^2 + \tau^2$, that is, depending on θ_1, smaller or larger than σ^2 (the variance of the error terms if there is no measurement error).

If we further assume that δ_2 is iid normally distributed, we can conclude that $\hat{\alpha}_{Y^*}$ is the MLE for $\theta_0 + \alpha_Y$ and $\hat{\beta}_{Y^*}$ is the MLE for $\theta_1 \beta_Y$. Conversely, $\hat{\beta}_{Y^*}$ is no longer the MLE for β_Y, if there is systematic measurement error in Y^*. In the absence of a treatment effect, as $\theta_1 \beta_Y = 0$ if $\beta_Y = 0$, T defined in (7) still follows a Student's t distribution with $N - 2$ degrees of freedom. In the presence of any given treatment effect, T follows a non-central Student's t distribution with $N - 2$ degrees of freedom and non-centrality parameter $(\theta_1 \beta_Y - \beta_0)/\sqrt{\widehat{\text{Var}}(\hat{\beta}_{Y^*})}$. Depending on the value of θ_1, the non-centrality parameter will be smaller or larger than the non-centrality parameter in the absence of measurement error (see section 3.2).

In summary, if there is systematic measurement error in the endpoints, the Type-I error is unaffected under standard regularity conditions and hence testing whether there is no effect is still valid under the null hypothesis [11]). Type-II, however, is affected (it may increase or decrease) and the treatment effect estimator is a biased MLE.

2.4 Differential measurement error

There is differential measurement error in Y^* when measurement error varies with X. Assuming a linear model for this variation, formally:

$$Y^* = \theta_{00} + (\theta_{01} - \theta_{00})X + \theta_{10} Y + (\theta_{11} - \theta_{10})XY + e_X, \tag{11}$$

where $\text{E}[e_X] = 0$ and $\text{Var}(e_X) = \tau_X^2$ and e_X independent of the endpoint of interest Y, and ε in (1). From the equations it becomes clear that systematic error (equation (10)) can be seen as a special case of differential error, where $\theta_{00} = \theta_{01}$ and $\theta_{10} = \theta_{11}$.

By using that $Y = \alpha_Y + \beta_Y X + \varepsilon$ from (1), it follows from equation (11) that,

$$Y^* = \theta_{00} + \theta_{10}\alpha_Y + \big[\theta_{01} - \theta_{00} + (\theta_{11} - \theta_{10})\alpha_Y + \theta_{11}\beta_Y\big]X + \big[\theta_{10} + (\theta_{11} - \theta_{10})X\big]\varepsilon + e_X.$$

Let $\delta_{3X} = \big[\theta_{10} + (\theta_{11} - \theta_{10})X\big]\varepsilon + e_X$, with expected variance $\big[\theta_{10}^2 + (\theta_{11}^2 - \theta_{10}^2)X\big]\sigma^2 + \tau_X^2$. Since the the error term δ_{3X} is no longer homoscedastic, the OLS estimators defined in (3) and (4) are no longer BLUE. However, the OLS estimator $\hat{\beta}_{Y^*}$ in (3) is consistent (although not efficient) for $\theta_{01} - \theta_{00} + (\theta_{11} - \theta_{10})\alpha_Y + \theta_{11}\beta_Y$. The OLS estimator $\hat{\alpha}_{Y^*}$ defined in (4) is consistent (although not efficient) for $\theta_{00} + \theta_{10}\alpha_Y$. Nevertheless, the estimator for the variance of $\hat{\beta}_{Y^*}$ defined in (8) is no longer valid.

By using the residuals ω_i defined in (6), a heteroscedastic consistent estimator for the variance of $\hat{\beta}_{Y^*}$ is:

$$\widehat{\mathrm{Var}}(\hat{\beta}_{Y^*}) = \frac{\sum_i \big[(X_i - \bar{X})^2\omega_i^2\big]}{[\sum_i (X_i - \bar{X})^2]^2},$$

which is known as the White estimator [12]. From standard regression theory, it is known that using the above defined estimator, T defined in (7) is still valid. Yet, under differential measurement error no longer $\big[\theta_{01} - \theta_{00} + (\theta_{11} - \theta_{10})\alpha_Y + \theta_{11}\beta_Y\big] = 0$ if $\beta_Y = 0$. Thus, under the null hypothesis, T defined in (7) follows a Student's t distribution with $N - 2$ degrees of freedom and non-centrality parameter $(\big[\theta_{01} - \theta_{00} + \theta_{11}\alpha_Y - \theta_{10}\alpha_Y + \theta_{11}\beta_0\big] - \beta_0)/\sqrt{\widehat{\mathrm{Var}}(\hat{\beta}_{Y^*})}$. Consequently, Type-I error changes if there is differential measurement error in Y^* and test about contrast under the null hypothesis are invalid [11]. Moreover, under the alternative hypothesis, T follows a non-central Student's t distribution with $N-2$ degrees of freedom and non-centrality parameter $(\big[\theta_{01} - \theta_{00} + (\theta_{11} - \theta_{10})\alpha_Y + \theta_{11}\beta_Y\big] - \beta_0)/\sqrt{\widehat{\mathrm{Var}}(\hat{\beta}_{Y^*})}$. Depending on the values of the θ's and α_Y, the non-centrality parameters will be smaller or larger than 0 and the non-centrality parameter if there is no measurement error, respectively (see section 3.2). Hence, Type-I error and Type-II error could increase or decrease if there is differential measurement error in Y^*.

To summarize, Type-I error is not expected nominal (α) if there is differential measurement error in Y^* (see also [11]). Also, similar to systematic error in Y^*, Type-II error is affected (may increase or decrease) and the treatment effect estimator is a biased estimator.

3 Correction methods for measurement error in continuous endpoints

To accommodate measurement error correction, we assume that Y and Y^* are both measured for a smaller set of different individuals not included in the trial ($j = 1, \dots, K, K < N$), hereinafter referred to as the external calibration sample. In all but one case, it is assumed that only Y^* and Y are measured in the external calibration sample. In the case that the error in Y^* is different for the two treatment groups, it is assumed that the external calibration sample is in the form of a small pilot study where both treatments are allocated (i.e., Y^* and Y are both measured after assignment of X).

3.1 Systematic measurement error

Using an external calibration set and assuming that the errors e in (10) are iid normal, the MLE of the measurement error parameters in (10) are:

$$\begin{aligned}\hat{\theta}_1 &= \frac{\sum_j (Y_j^{(c)} - \bar{Y}^{(c)})(Y_j^{*(c)} - \bar{Y}^{*(c)})}{\sum (Y_j^{(c)} - \bar{Y}^{(c)})^2}, \\ \hat{\theta}_0 &= \bar{Y}^{*(c)} - \hat{\theta}_1 \bar{Y}^{(c)}, \\ t^2 &= \frac{1}{K-2} \sum_j (Y_j^{*(c)} - \hat{\theta}_0 - \hat{\theta}_1 Y_j^{(c)})^2.\end{aligned} \tag{12}$$

The superscript (c) is used to indicate that the measurement is obtained in the calibration set. From section 3.4, under systematic measurement error and assuming that ε in (1) and e in (10) iid normal and independent, the estimator $\hat{\beta}_{Y^*}$ defined in (3) is the MLE of $\theta_1\beta_Y$ and, the estimator $\hat{\alpha}_{Y^*}$ defined in (4) is the MLE of $\theta_0 + \theta_1\alpha_Y$. Natural sample estimators for α_Y and β_Y are then

$$\hat{\alpha}_Y = (\hat{\alpha}_{Y^*} - \hat{\theta}_0)/\hat{\theta}_1 \quad \text{and} \quad \hat{\beta}_Y = \hat{\beta}_{Y^*}/\hat{\theta}_1, \tag{13}$$

where $\hat{\theta}_0$ and $\hat{\theta}_1$ are the estimated error parameters from the calibration data set. From equation (13), it becomes apparent that $\hat{\theta}_1$ needs to be assumed bounded away from zero for finite estimates of $\hat{\alpha}_Y$ and $\hat{\beta}_Y$ [13].
The first moment of estimators $\hat{\alpha}_Y$ and $\hat{\beta}_Y$ can be approximated by using multivariate Taylor expansions and assuming that ($\hat{\alpha}_{Y^*}$, $\hat{\beta}_{Y^*}$, $\hat{\theta}_0$, $\hat{\theta}_1$) are normally distributed [13],

$$\mathrm{E}[\hat{\alpha}_Y] \approx \alpha_Y + \frac{[\alpha_Y - \bar{y^*}]\tau^2}{\theta_1^2 S_{yy}^{(c)}} \quad \text{and} \quad \mathrm{E}[\hat{\beta}_Y] \approx \beta_Y + \frac{\beta_Y \tau^2}{\theta_1^2 S_{yy}^{(c)}},$$

where $S_{yy}^{(c)} = \sum (Y_j^{(c)} - \bar{Y}^{(c)})^2$, the total sum of squares of $Y^{(c)}$. In conclusion, the estimators $\hat{\alpha}_Y$ and $\hat{\beta}_Y$ are consistent. Formal derivations for the presented formulas are provided in the Appendix.

In the following we will focus on specifying confidence limits for the treatment effect estimator $\hat{\beta}_Y$ defined in (13). We make use of the fact that this estimator is a ratio, which motivates the use of the Delta method, Fieller method and Zero-variance method [14]. We also present a non-parametric bootstrap method for specifying confidence limits [15].

3.1.1 Delta method

Assuming that $\hat{\beta}_{Y^*}$ and $\hat{\theta}_1$ are both normally distributed and applying the Delta method, the second moment of $\hat{\beta}_Y$ can be approximated [11]. Formal derivations of the presented formulas are provided in Appendix A. The Delta method variance of $\hat{\beta}_Y$ is given by:

$$\mathrm{Var}(\hat{\beta}_Y) \approx \frac{1}{\theta_1^2}\Big[\frac{\theta_1^2\sigma^2 + \tau^2}{S_{xx}} + \frac{\beta_Y^2\tau^2}{S_{yy}^{(c)}}\Big],$$

where $S_{xx} = \sum_i (X_i - \bar{X})^2$, the total sum of squares of X. An approximation of the above defined variance, denoted by $\widehat{\mathrm{Var}}(\hat{\beta}_Y)$, is provided by approximating θ_1, $\theta_1^2\sigma^2 + \tau^2$, τ^2 and β_Y respectively by $\hat{\theta}_1$, s^2, t^2 and $\hat{\beta}_Y$ [11].

An approximate confidence interval for the estimator $\hat{\beta}_Y$ is then given by

$$\hat{\beta}_Y \pm t_{(\alpha/2,n-2)}\sqrt{\widehat{\text{Var}}\big(\hat{\beta}_Y\big)}. \tag{14}$$

3.1.2 Fieller method

A second method to construct confidence intervals for the estimator $\hat{\beta}_Y$ in (13), described by Buonaccorsi, is the Fieller method [11, 16]. In the case that $\hat{\theta}_1$ is significantly different from zero at a significance level of α (that is, $\hat{\theta}_1/\sqrt{t^2/S_{yy}^{(c)}} > t_{N-2}$), the $(1-\alpha)$ confidence intervals of $\hat{\beta}_Y$ are defined by the Fieller method by:

$$l_{upper,lower} = \frac{\hat{\beta}_{Y^*}\hat{\theta}_1 \pm \sqrt{\hat{\beta}^2_{Y^*}\hat{\theta}^2_1 - (\frac{t^2}{S_{yy}^{(c)}}t_q^2 - \hat{\theta}^2_1)(\frac{s^2}{S_{xx}}t_q^2 - \hat{\beta}^2_{Y^*})}}{\frac{\tau^2}{S_{yy}^{(c)}}t_q^2 + \hat{\theta}_1^2}. \tag{15}$$

A formal derivation can be found in Appendix A.

3.1.3 Zero-variance method

The zero-variance method adjusts the observed endpoints Y_i^* by

$$\hat{Y}_i = (Y_i^* - \hat{\theta}_0)/\hat{\theta}_1,$$

where $\hat{\theta}_0$ and $\hat{\theta}_1$ are derived from (10). The adjusted endpoints are regressed on the treatment variable X, which yields,

$$\begin{aligned}
\hat{\beta}_{\hat{Y}} &= \frac{\sum_i (X_i - \bar{X})(\hat{Y}_i - \bar{\hat{Y}})}{\sum_i (X_i - \bar{X})^2} = \frac{\sum_i (X_i - \bar{X})(Y_i^* - \bar{Y^*})/\hat{\theta}_1}{\sum_i (X_i - \bar{X})^2} = \hat{\beta}_{Y^*}/\hat{\theta}_1,\\
\hat{\alpha}_{\hat{Y}} &= \bar{\hat{Y}} - \hat{\beta}_{\hat{Y}}\bar{X} = \frac{\bar{Y^*} - \hat{\beta}_{Y^*}\bar{X} - \hat{\theta}_0}{\hat{\theta}_1} = (\hat{\alpha}_{Y^*} - \hat{\theta}_0)/\hat{\theta}_1,\\
s^2_{\hat{Y}} &= \frac{1}{N-2}\sum_i (\hat{Y}_i - \hat{\alpha}_{\hat{Y}} - \hat{\beta}_{\hat{Y}}X_i)^2 = \frac{1}{\hat{\theta}_1^2}s^2,
\end{aligned}$$

with $\hat{\beta}_{Y^*}$, $\hat{\alpha}_{Y^*}$ and s^2 as in equations (3, 4 and 6), respectively. Thus, $\hat{\beta}_{\hat{Y}}$ equals $\hat{\beta}_Y$ and $\hat{\alpha}_{\hat{Y}}$ equals $\hat{\alpha}_Y$ defined in (13).

If the value of $\hat{\theta}_1$ (i.e. θ_1) is known, the variance of the estimator $\hat{\beta}_{\hat{Y}}$ is equal to:

$$\text{Var}(\hat{\beta}_{\hat{Y}}) = \text{Var}(\hat{\beta}_{Y^*})/\theta_1^2 = \frac{\sigma^2 + \tau^2/\theta_1^2}{\sum_i (X_i - \bar{X})^2}.$$

Using the standard OLS regression framework the variance of $\hat{\beta}_{\hat{Y}}$ can be estimated by:

$$\widehat{\text{Var}}(\hat{\beta}_{\hat{Y}}) = \frac{s^2_{\hat{Y}}}{\sum_i (X_i - \bar{X})^2} = \frac{s^2/\hat{\theta}_1^2}{\sum_i (X_i - \bar{X})^2}. \tag{16}$$

By replacing $\hat{\theta}_1$ by θ_1 in the above, the quantity in (16) is in expectation equal to Var($\hat{\beta}_{\hat{Y}}$) (defined above). The quantity in (16) is used in the zero-variance method to construct confidence intervals for $\hat{\beta}_{\hat{Y}}$, by replacing $\widehat{\text{Var}}(\hat{\beta}_{\hat{Y}})$ for $\widehat{\text{Var}}(\hat{\beta}_Y)$ in equation 14. In conclusion, this zero-variance approach will provide confidence intervals for the treatment effect estimator while assuming there is no variance in $\hat{\theta}_1$ (giving it its name zero-variance method). Although the zero-variance approach wins in terms of simplicity, it may underestimate the variability of the ratio since the variance in $\hat{\theta}_1$ is assumed zero.

3.1.4 Bootstrap

An alternative for defining confidence intervals for the corrected treatment effect estimator $\hat{\beta}_Y$ is by using a non-parametric bootstrap [15]. We propose the following stepwise procedure:

1. Draw a random sample with replacement of size K of the calibration sample $(Y^{*(c)}, Y^{(c)})$ to estimate $\hat{\theta}_{1_B}$ defined in (12).
2. Draw a random sample with replacement of size N of the trial data (Y^*, X) to calculate the corrected treatment effect estimate by $\hat{\beta}_{Y_B} = \beta_{Y^*_B}/\hat{\theta}_{1_B}$. Where $\beta_{Y^*_B}$ is defined in (3).
3. Repeat step 1-2 B times, with B large (e.g. 999 times).
4. Approximate confidence intervals are given by the $(\alpha/2, 1-\alpha/2)$ percentile of the distribution of $\hat{\beta}_{Y_B}$.

3.2 Differential measurement error

For corrections for endpoints that suffer from differential measurement error we will here assume the existence of a pilot trial, which serves as an external calibration set, where both treatments are allocated at random that serves as an external calibration set to estimate the measurement error model in (11). For notational convenience we rewrite the linear model in equation (11) in matrix form as:

$$\boldsymbol{Y}^* = \boldsymbol{X}\boldsymbol{\theta} + \boldsymbol{e}, \tag{17}$$

where E($\boldsymbol{e}$) $= 0$ and E($\boldsymbol{e}\boldsymbol{e}'$) $= \boldsymbol{\Sigma}$, a positive definite matrix, with τ_X^2 on its diagonal. Further, $\boldsymbol{\theta} = (\theta_1, \theta_2, \theta_3, \theta_4) = (\theta_{00}, \theta_{01} - \theta_{00}, \theta_{10}, \theta_{11} - \theta_{10})$. In the external calibration set, the measurement error parameters $\hat{\boldsymbol{\theta}}$ can be estimated by,

$$\hat{\boldsymbol{\theta}} = (\boldsymbol{X}^{(c)\prime}\boldsymbol{X}^{(c)})^{-1}\boldsymbol{X}^{(c)\prime}\boldsymbol{Y}^{(c)}, \tag{18}$$

with variance,

$$\text{Var}(\hat{\boldsymbol{\theta}}) = (\boldsymbol{X}^{(c)\prime}\boldsymbol{X}^{(c)})^{-1}\boldsymbol{X}^{(c)\prime}\Sigma\boldsymbol{X}^{(c)}(\boldsymbol{X}^{(c)\prime}\boldsymbol{X}^{(c)})^{-1}.$$

See [12] for a discussion on different estimators for the above defined variance. From section 2.5 it follows that natural estimators for α_Y and β_Y are,

$$\hat{\alpha}_Y = (\hat{\alpha}_{Y^*} - \hat{\theta}_{00})/\hat{\theta}_{10} \quad \text{and} \quad \hat{\beta}_Y = (\hat{\beta}_{Y^*} + \hat{\alpha}_{Y^*} - \hat{\theta}_{01})/\hat{\theta}_{11} - \hat{\alpha}_Y, \tag{19}$$

where $\hat{\theta}_{00}$, $\hat{\theta}_{10}$, $\hat{\theta}_{01}$ and $\hat{\theta}_{11}$ are estimated from the external calibration set. Here it is assumed that both $\hat{\theta}_{10}$ and $\hat{\theta}_{11}$ are bounded away from zero (for reasons similar to those mentioned in section 3.1).

By multivariate Taylor expansions, the first moments of the estimators $\hat{\alpha}_Y$ and $\hat{\beta}_Y$ defined in (19)

can be approximated [11], in the same way as the estimators for systematic measurement error (section 4.1),

$$\mathrm{E}\big[\hat{\alpha}_Y\big] \approx \alpha_Y + \frac{1}{\theta_{10}^2}\Big[\alpha_Y \mathrm{Var}\big(\hat{\theta}_{10}\big) + \mathrm{Cov}\big(\hat{\theta}_{00}, \hat{\theta}_{10}\big)\Big],$$

$$\mathrm{E}\big[\hat{\beta}_Y\big] \approx \beta_Y + \frac{1}{\theta_{11}^2}\Big[(\beta_Y + \alpha_Y)\mathrm{Var}\big(\hat{\theta}_{11}\big) + \mathrm{Cov}\big(\hat{\theta}_{01}, \hat{\theta}_{11}\big)\Big] - \frac{1}{\theta_{10}^2}\Big[\alpha_Y \mathrm{Var}\big(\hat{\theta}_{10}\big) + \mathrm{Cov}\big(\hat{\theta}_{00}, \hat{\theta}_{10}\big)\Big].$$

From this, it is apparent that the estimators $\hat{\alpha}_Y$ and $\hat{\beta}_Y$ defined in (19) are consistent (details are found in the Appendix). In the subsequent sections we review the Delta method, zero-variance and propose a bootstrap for specifying confidence limits for the estimator of the treatment effect under differential measurement error of the endpoints.

3.2.1 Delta method

The variance of the estimator $\hat{\beta}_Y$ defined in (19) can be approximated by the Delta method [11]:

$$\mathrm{Var}\big(\hat{\beta}_Y\big) \approx \frac{1}{\theta_{11}^2}\Big[\big(\beta_Y + \alpha_Y\big)^2 \mathrm{Var}\big(\hat{\theta}_{11}\big) + \mathrm{Var}\big(\hat{\beta}_{Y^*}\big) + \mathrm{Var}\big(\hat{\alpha}_{Y^*}\big) + 2\mathrm{Cov}\big(\hat{\alpha}_{Y^*}, \hat{\beta}_{Y^*}\big) + \mathrm{Var}\big(\hat{\theta}_{01}\big) + 2\big(\beta_Y + \alpha_Y\big)\mathrm{Cov}\big(\hat{\theta}_{11}, \hat{\theta}_{01}\big)\Big] + \mathrm{Var}\big(\hat{\alpha}_Y\big),$$

where $\mathrm{Var}\big(\hat{\alpha}_Y\big)$ is approximated by:

$$\mathrm{Var}\Big(\frac{\hat{\alpha}_{Y^*} - \hat{\theta}_{00}}{\hat{\theta}_{10}}\Big) \approx \frac{1}{\theta_{10}^2}\Big[\mathrm{Var}\big(\hat{\alpha}_{Y^*}\big) + \alpha_Y^2 \mathrm{Var}\big(\hat{\theta}_{10}\big) + \mathrm{Var}\big(\hat{\theta}_{00}\big) + 2\alpha_Y \mathrm{Cov}\big(\hat{\theta}_{00}, \hat{\theta}_{10}\big)\Big].$$

An approximate confidence interval for the estimator $\hat{\beta}_Y$ in (19) is:

$$\hat{\beta}_Y \pm t_{(\alpha/2, n-2)} \sqrt{\mathrm{Var}\big(\hat{\beta}_Y\big)}. \tag{20}$$

An approximation of θ_{11}, θ_{10}, $\theta_{11}^2\sigma^2 + \tau_1^2$, $\theta_{10}^2\sigma^2 + \tau_0^2$, τ_1^2, τ_0^2, β_Y and α_Y in the above is provided by: $\hat{\theta}_{11}$, $\hat{\theta}_{10}$, s_1^2, s_0^2, t_1^2, t_0^2, $\hat{\beta}_Y$ and $\hat{\alpha}_Y$ [11].

3.2.2 Zero-variance method

The zero-variance method adjusts the observed endpoints Y_i^* by

$$\hat{Y}_{ix} = (Y_{ix}^* - \hat{\theta}_{0x})/\hat{\theta}_{1x},$$

for $x \in \{0, 1\}$ and $\hat{\theta}_{0x})$ and $\hat{\theta}_{1x}$ derived from (18). In the zero-variance method the above defined adjusted values are regressed on the treatment variable X, yielding in estimators $\hat{\alpha}_{\hat{Y}}$ and $\hat{\beta}_{\hat{Y}}$, which are, respectively, equal to the estimators $\hat{\alpha}_Y$ and $\hat{\beta}_Y$ defined in (19). The variance of these estimators can be approximated with a heteroscedastic consistent covariance estimator (see [12] for an overview). Confidence intervals for $\hat{\beta}_{\hat{Y}}$ are subsequently constructed by using formula 20. Similar to what is described in section 4.1.3 discussing the zero-variance method for systematic measurement error, this way of constructing confidence intervals neglects the variance of the θ's from the calibration data set, and will thus often yield in confidence intervals that are too narrow.

3.2.3 Bootstrap

We here alternatively propose a non-parametric bootstrap procedure to specify confidence limits. This entails the following steps:

1. Draw a random sample with replacement of size K of the calibration sample and estimate $\hat{\boldsymbol{\theta}}$ as defined in (18).
2. Draw a random sample (with replacement) of size N of the study population and calculate the effect estimate by $\hat{\alpha}_{Y_B} = (\alpha_{Y^*_B} - \hat{\theta}_{00_B})/\hat{\theta}_{10_B}$ and $\hat{\beta}_{Y_B} = (\beta_{Y^*_B} + \alpha_{Y^*_B} - \hat{\theta}_{01_B})/\hat{\theta}_{11_B} - \hat{\alpha}_{Y_B}$. Where $\beta_{Y^*_B}$ and $\alpha_{Y^*_B}$ are defined in (3) and (4), respectively.
3. Repeat step 1-2 B times, with B large (e.g. 999 times).
4. Approximate confidence intervals are given by the $(\alpha/2, 1-\alpha/2)$ percentile of the distribution of $\hat{\beta}_{Y_B}$.

4 Measurement error depending on prognostic factors

Suppose that there is a prognostic factor S, and assume that, $\mathrm{E}[Y|X,S] = \alpha_Y + \beta_Y X + \gamma_Y S$, $\mathrm{E}[Y^*|Y,S] = Y + \zeta S$, $Y^* \perp\!\!\!\perp X|Y$ (non-differential measurement error) and $S \perp\!\!\!\perp X$ (randomization is well-performed).

Suppose we want to estimate the effect of Y on X (i.e., β_Y), but instead of Y we have only measured the with measurement error contaminated Y^*. If one is aware that there is a prognostic factor that confounds the relation between Y^* and Y (and this factor is measured), one could decide to regress Y^* on X and S. The regression of Y^* on X and S equals,

$$\begin{aligned}
\mathrm{E}[Y^*|X,S] &= \mathrm{E}_{Y|X,S}\{\mathrm{E}_{\mathrm{Y^*|X,S,Y}}[\mathrm{Y^*|X,S,Y}]|\mathrm{X,S}\} \\
&= \mathrm{E}_{Y|X,S}\{\mathrm{E}_{\mathrm{Y^*|S,Y}}[\mathrm{Y^*|S,Y}]|\mathrm{X,S}\} \\
&= \mathrm{E}_{Y|X,S}\{\mathrm{Y} + \zeta\mathrm{S}|\mathrm{X,S}\} \\
&= \alpha_Y + \beta_Y X + (\gamma_Y + \zeta)S.
\end{aligned}$$

Thus, using the with measurement error contaminated endpoint Y^* instead of the preferred endpoint Y will provide an unbiased estimation of β_Y.

However, if one is not aware of the prognostic factor, one might naively regress Y^* on X, which equals:

$$\begin{aligned}
\mathrm{E}[Y^*|X] &= \mathrm{E}_{S|X}\{\mathrm{E}_{Y|X,S}\{\mathrm{E}_{\mathrm{Y^*|X,S,Y}}[\mathrm{Y^*|X,S,Y}]|\mathrm{X,S}\}|X\} \\
&= \mathrm{E}_{S|X}\{\alpha_Y + \beta_Y X + (\gamma_Y + \zeta)S|X\} \\
&= \alpha_Y + \beta_Y X + (\gamma_Y + \zeta)\mathrm{E}[S].
\end{aligned}$$

In conclusion, by ignoring the prognostic factor and using the with measurement error contaminated endpoint Y^* instead of the preferred endpoint Y, the regression of Y^* on X still results in an unbiased estimation of β_Y.

A1 Approximation of bias and variance in corrected estimator

A1.1 Systematic measurement error

Obvious estimators for α_Y and β_Y are:

$$\hat{\alpha}_Y = (\hat{\alpha}_{Y^*} - \hat{\theta}_0)/\hat{\theta}_1 \quad \text{and} \quad \hat{\beta}_Y = \hat{\beta}_{Y^*}/\hat{\theta}_1.$$

These estimators can be approximated with a second order Taylor expansion by:

$$\begin{aligned}
\frac{\hat{\beta}_{Y^*}}{\hat{\theta}_1} &\approx \frac{\beta_{Y^*}}{\theta_1} - \frac{\beta_{Y^*}}{\theta_1^2}(\hat{\theta}_1 - \theta_1) + \frac{1}{\theta_1}(\hat{\beta}_{Y^*} - \beta_{Y^*}) \\
&+ \frac{1}{2!}\Big[\frac{2\beta_{Y^*}}{\theta_1^3}(\hat{\theta}_1 - \theta_1)^2 - \frac{2}{\theta_1^2}(\hat{\theta}_1 - \theta_1)(\hat{\beta}_{Y^*} - \beta_{Y^*})\Big], \\
\frac{\hat{\alpha}_{Y^*}}{\hat{\theta}_1} &\approx \frac{\alpha_{Y^*}}{\theta_1} - \frac{\alpha_{Y^*}}{\theta_1^2}(\hat{\theta}_1 - \theta_1) + \frac{1}{\theta_1}(\hat{\alpha}_{Y^*} - \alpha_{Y^*}) \\
&+ \frac{1}{2!}\Big[\frac{2\alpha_{Y^*}}{\theta_1^3}(\hat{\theta}_1 - \theta_1)^2 - \frac{2}{\theta_1^2}(\hat{\theta}_1 - \theta_1)(\hat{\alpha}_{Y^*} - \alpha_{Y^*})\Big], \\
\frac{\hat{\theta}_0}{\hat{\theta}_1} &\approx \frac{\theta_0}{\theta_1} - \frac{\theta_0}{\theta_1^2}(\hat{\theta}_1 - \theta_1) + \frac{1}{\theta_1}(\hat{\theta}_0 - \theta_0) \\
&+ \frac{1}{2!}\Big[\frac{2\theta_0}{\theta_1^3}(\hat{\theta}_1 - \theta_1)^2 - \frac{2}{\theta_1^2}(\hat{\theta}_1 - \theta_1)(\hat{\theta}_0 - \theta_0)\Big].
\end{aligned}$$

Simplifying these terms and substraction of the latter two, will lead to the following approximations for $\hat{\alpha}_Y$ and $\hat{\beta}_Y$:

$$\begin{aligned}
\frac{\hat{\beta}_{Y^*}}{\hat{\theta}_1} &\approx \frac{\beta_{Y^*}}{\theta_1} + \frac{1}{\theta_1}\Big[-\frac{\beta_{Y^*}}{\theta_1}(\hat{\theta}_1 - \theta_1) + (\hat{\beta}_{Y^*} - \beta_{Y^*})\Big] \\
&+ \frac{1}{\theta_1^2}\Big[\frac{\beta_{Y^*}}{\theta_1}(\hat{\theta}_1 - \theta_1)^2 - (\hat{\beta}_{Y^*} - \beta_{Y^*})(\hat{\theta}_1 - \theta_1)\Big], \\
\frac{\hat{\alpha}_{Y^*} - \hat{\theta}_0}{\hat{\theta}_1} &\approx \frac{\alpha_{Y^*} - \theta_0}{\theta_1} + \frac{1}{\theta_1}\Big[-\frac{\alpha_{Y^*} - \theta_0}{\theta_1}(\hat{\theta}_1 - \theta_1) + (\hat{\alpha}_{Y^*} - \alpha_{Y^*}) - (\hat{\theta}_0 - \theta_0)\Big] \\
&+ \frac{1}{\theta_1^2}\Big[\frac{\alpha_{Y^*} - \theta_0}{\theta_1}(\hat{\theta}_1 - \theta_1)^2 - (\hat{\alpha}_{Y^*} - \alpha_{Y^*})(\hat{\theta}_1 - \theta_1) + (\hat{\theta}_0 - \theta_0)(\hat{\theta}_1 - \theta_1)\Big].
\end{aligned}$$

Since $\mathrm{E}[\hat{\theta}_1 - \theta_1] = 0$, $\mathrm{E}[\hat{\theta}_0 - \theta_0] = 0$, $\mathrm{E}[\hat{\alpha}_{Y^*} - \alpha_{Y^*}] = 0$ and $\mathrm{E}[\hat{\beta}_{Y^*} - \beta_{Y^*}] = 0$ an approximation of the expected value of the estimator $\hat{\alpha}_Y$ is given by:

$$\begin{aligned}
\mathrm{E}\Big[\frac{\hat{\alpha}_{Y^*} - \hat{\theta}_0}{\hat{\theta}_1}\Big] &\approx \frac{\alpha_{Y^*} - \theta_0}{\theta_1} + \frac{1}{\theta_1^2}\Big[\frac{\alpha_{Y^*} - \theta_0}{\theta_1}\mathrm{E}\big[(\hat{\theta}_1 - \theta_1)^2\big] \\
&- \mathrm{E}\big[(\hat{\alpha}_{Y^*} - \alpha_{Y^*})(\hat{\theta}_1 - \theta_1)\big] + \mathrm{E}\big[(\hat{\theta}_0 - \theta_0)(\hat{\theta}_1 - \theta_1)\big]\Big] = \\
&= \frac{\alpha_{Y^*} - \theta_0}{\theta_1} + \frac{1}{\theta_1^2}\Big[\frac{\alpha_{Y^*} - \theta_0}{\theta_1}\mathrm{Var}\big(\hat{\theta}_1\big) - \mathrm{Cov}\big(\hat{\alpha}_{Y^*}, \hat{\theta}_1\big) + \mathrm{Cov}\big(\hat{\theta}_0, \hat{\theta}_1\big)\Big] = \\
&= \alpha_Y + \frac{1}{\theta_1^2}\Big[\frac{\tau^2[\alpha_Y - \bar{Y}^{(c)}]}{\sum(Y_j^{(c)} - \bar{Y}^{(c)})^2}\Big].
\end{aligned}$$

Congruently, an approximation of the expected value of the estimator $\hat{\beta}_Y$ is given by:

$$\begin{aligned}
\mathrm{E}\Big[\frac{\hat{\beta}_{Y^*}}{\hat{\theta}_1}\Big] &\approx \frac{\beta_{Y^*}}{\theta_1} + \frac{1}{\theta_1^2}\Big[\frac{\beta_{Y^*}}{\theta_1}\mathrm{E}\big[(\hat{\theta}_1-\theta_1)^2\big] - \mathrm{E}\big[(\hat{\beta}_{Y^*}-\beta_{Y^*})(\hat{\theta}_1-\theta_1)\big]\Big] = \\
&= \frac{\beta_{Y^*}}{\theta_1} + \frac{1}{\theta_1^2}\Big[\frac{\beta_{Y^*}}{\theta_1}\mathrm{Var}\big(\hat{\theta}_1\big)\Big] = \\
&= \beta_Y + \frac{1}{\theta_1^2}\Big[\frac{\tau^2\beta_Y}{\sum(Y_j^{(c)}-\bar{Y}^{(c)})^2}\Big].
\end{aligned}$$

Only using the first order Taylor expansion of the estimators, approximations of the variance of $\hat{\alpha}_Y$ and $\hat{\beta}_Y$ are respectively:

$$\begin{aligned}
\mathrm{Var}\big(\frac{\hat{\alpha}_{Y^*}-\hat{\theta}_0}{\hat{\theta}_1}\big) &\approx \frac{1}{\theta_1^2}\Big[\alpha_Y^2\mathrm{Var}\big(\hat{\theta}_1\big) + \mathrm{Var}\big(\hat{\alpha}_{Y^*}-\hat{\theta}_0\big) - 2\alpha_Y\mathrm{Cov}\big(\hat{\theta}_1,\hat{\alpha}_{Y^*}-\hat{\theta}_0\big)\Big] = \\
&= \frac{1}{\theta_1^2}\Big[\alpha_Y^2\mathrm{Var}\big(\hat{\theta}_1\big) + \mathrm{Var}\big(\hat{\alpha}_{Y^*}\big) + \mathrm{Var}\big(\hat{\theta}_0\big) - 2\mathrm{Cov}\big(\hat{\alpha}_{Y^*},\hat{\theta}_0\big) \\
&- 2\alpha_Y\mathrm{Cov}\big(\hat{\theta}_1,\hat{\alpha}_{Y^*}\big) + 2\alpha_Y\mathrm{Cov}\big(\hat{\theta}_1,\hat{\theta}_0\big)\Big] = \\
&= \frac{1}{\theta_1^2}\Big[\frac{(\theta_1^2\sigma^2+\tau^2)\sum X_i^2}{N\sum(X_i-\bar{X})^2} + \alpha_Y^2\frac{\tau^2}{\sum(Y_j^{(c)}-\bar{Y}^{(c)})^2} + \frac{\tau^2\sum(Y_j^{(c)})^2}{K\sum(Y_j^{(c)}-\bar{Y}^{(c)})^2} \\
&+ 2\alpha_Y\frac{-\tau^2\bar{Y}^{(c)}}{\sum(Y_j^{(c)}-\bar{Y}^{(c)})^2}\Big] = \\
&= \frac{1}{\theta_1^2}\Big[\frac{(\theta_1^2\sigma^2+\tau^2)\sum X_i^2}{N\sum(X_i-\bar{x})^2} + \alpha_Y^2\frac{\tau^2}{\sum(y_j^{(c)}-\bar{y}^{(c)})^2} + \frac{\tau^2(\sum(y_j^{(c)}-\bar{y}^{(c)})^2+K(\bar{y}^{(c)})^2)}{K\sum(y_j^{(c)}-\bar{y}^{(c)})^2} \\
&- 2\alpha_Y\frac{\tau^2\bar{y}^{(c)}}{\sum(y_j^{(c)}-\bar{y}^{(c)})^2}\Big] = \\
&= \frac{1}{\theta_1^2}\Big[\frac{(\theta_1^2\sigma^2+\tau^2)\sum x_i^2}{N\sum(x_i-\bar{x})^2} + \tau^2\Big(\frac{1}{K} + \frac{(\bar{y}^{(c)}-\alpha_Y)^2}{\sum(y_j^{(c)}-\bar{y}^{(c)})^2}\Big)\Big], \\
\mathrm{Var}\big(\frac{\hat{\beta}_{Y^*}}{\hat{\theta}_1}\big) &\approx \frac{1}{\theta_1^2}\Big[\frac{\theta_1^2\sigma^2+\tau^2}{\sum(x_i-\bar{x})^2} + \frac{\beta_Y^2\tau^2}{\sum(y_j^{(c)}-\bar{y}^{(c)})^2}\Big].
\end{aligned}$$

A1.1.1 Fieller method

Assume that $\hat{\beta}_{Y^*}$ and $\hat{\theta}_1$ are normally distributed (note that this assumption is satisfied with large study samples (N) and large calibration samples (K)). The sum of two normally distributed variables is normally distributed, hence, $\hat{\beta}_{Y^*} - \beta_Y\hat{\theta}_1$ is normally distributed.
Furthermore, we have

$$\mathrm{Var}(\hat{\beta}_{Y^*} - \beta_Y\hat{\theta}_1) = \mathrm{Var}(\hat{\beta}_{Y^*}) + \beta_Y^2\mathrm{Var}(\hat{\theta}_1).$$

Where,

$$\begin{aligned}\text{Var}(\hat{\beta}_{Y^*}) &= \frac{\theta_1^2\sigma^2+\tau^2}{\sum(x_i-\bar{x})^2}\\ \text{Var}(\hat{\theta}_1) &= \frac{\tau^2}{\sum(y_j^{(c)}-\bar{y}^{(c)})^2}\end{aligned}$$

If we now divide the term $\hat{\beta}_{Y^*}-\beta_Y\hat{\theta}_1$ by its standard deviation, we get:

$$T_0=\frac{\hat{\beta}_{Y^*}-\beta_Y\hat{\theta}_1}{\sqrt{\frac{\theta_1^2\sigma^2+\tau^2}{\sum(x_i-\bar{x})^2}+\frac{\tau^2}{\sum(y_j^{(c)}-\bar{y}^{(c)})^2}\beta_Y^2}} \tag{A1}$$

We are interested to find the set of β_Y values for which the corresponding T_0 values lie within the $(1-\alpha)$ quantiles of the t-distribution with $N-2$ degrees of freedom (this only holds approximately, see for details [14]). Let us denote these values by t_q, from (A1) we have,

$$\left(\frac{\tau^2}{\sum(y_j^{(c)}-\bar{y}^{(c)})^2}t_q^2-\hat{\theta}_1^2\right)\beta_Y^2+2\hat{\beta}_{Y^*}\hat{\theta}_1\beta_Y+\left(\frac{\theta_1^2\sigma^2+\tau^2}{\sum(x_i-\bar{x})^2}t_q^2-\hat{\beta}_{Y^*}^2\right)=0.$$

In the case that $\hat{\theta}_1$ is significantly different from zero at a significance level of α (that is, $\hat{\theta}_1/\sqrt{\frac{\tau^2}{\sum(y_j^{(c)}-\bar{y}^{(c)})^2}}>t_q$), solving this for β_Y results in the following $(1-\alpha)$ confidence intervals:

$$\beta_Y=\frac{-\hat{\beta}_{Y^*}\hat{\theta}_1\pm\sqrt{\hat{\beta}_{Y^*}^2\hat{\theta}_1^2-\left(\frac{\tau^2}{\sum(y_j^{(c)}-\bar{y}^{(c)})^2}t_q^2-\hat{\theta}_1^2\right)\left(\frac{\theta_1^2\sigma^2+\tau^2}{\sum(x_i-\bar{x})^2}t_q^2-\hat{\beta}_{Y^*}^2\right)}}{\frac{\tau^2}{\sum(y_j^{(c)}-\bar{y}^{(c)})^2}t_q^2-\hat{\theta}_1^2}.$$

In the other case, the confidence intervals are unbounded, see for more details [14].

A1.2 Differential measurement error

Obvious estimators for α_Y and β_Y are:

$$\hat{\alpha}_Y=(\hat{\alpha}_{Y^*}-\hat{\theta}_{00})/\hat{\theta}_{10}\quad\text{and}\quad\hat{\beta}_Y=(\hat{\beta}_{Y^*}+\hat{\alpha}_{Y^*}-\hat{\theta}_{01})/\hat{\theta}_{11}-\hat{\alpha}_Y.$$

These estimators can be approximated with a second order Taylor expansion by:

$$\begin{aligned}\frac{\hat{\alpha}_{Y^*}-\hat{\theta}_{00}}{\hat{\theta}_{10}}&\approx\frac{\alpha_{Y^*}-\theta_{00}}{\theta_{10}}+\frac{1}{\theta_{10}}\Big[-\frac{\alpha_{Y^*}-\theta_{00}}{\theta_{10}}(\hat{\theta}_{10}-\theta_{10})+(\hat{\alpha}_{Y^*}-\alpha_{Y^*})-(\hat{\theta}_{00}-\theta_{00})\Big]\\&\quad+\frac{1}{\theta_{11}^2}\Big[\frac{\alpha_{Y^*}-\theta_{00}}{\theta_{10}}(\hat{\theta}_{10}-\theta_{10})^2-(\hat{\alpha}_{Y^*}-\alpha_{Y^*})(\hat{\theta}_{10}-\theta_{10})+(\hat{\theta}_{00}-\theta_{00})(\hat{\theta}_{10}-\theta_{10})\Big],\\ \frac{\hat{\beta}_{Y^*}-\hat{\theta}_{01}}{\hat{\theta}_{11}}&\approx\frac{\beta_{Y^*}-\theta_{01}}{\theta_{11}}+\frac{1}{\theta_{11}}\Big[-\frac{\beta_{Y^*}-\theta_{01}}{\theta_{11}}(\hat{\theta}_{11}-\theta_{11})+(\hat{\beta}_{Y^*}-\beta_{Y^*})-(\hat{\theta}_{01}-\theta_{01})\Big]\\&\quad+\frac{1}{\theta_{11}^2}\Big[\frac{\beta_{Y^*}-\theta_{01}}{\theta_{11}}(\hat{\theta}_{11}-\theta_{11})^2-(\hat{\beta}_{Y^*}-\beta_{Y^*})(\hat{\theta}_{11}-\theta_{11})+(\hat{\theta}_{01}-\theta_{01})(\hat{\theta}_{11}-\theta_{11})\Big],\\ \frac{\hat{\alpha}_{Y^*}}{\hat{\theta}_{11}}&\approx\frac{\alpha_{Y^*}}{\theta_{11}}+\frac{1}{\theta_{11}}\Big[-\frac{\alpha_{Y^*}}{\theta_{11}}(\hat{\theta}_{11}-\theta_{11})+(\hat{\alpha}_{Y^*}-\alpha_{Y^*}))\Big]\\&\quad+\frac{1}{\theta_{11}^2}\Big[\frac{\alpha_{Y^*}}{\theta_{11}}(\hat{\theta}_{11}-\theta_{11})^2-(\hat{\alpha}_{Y^*}-\alpha_{Y^*})(\hat{\theta}_{11}-\theta_{11})\Big].\end{aligned}$$

Congruent to the results for the estimators under systematic measurement error, we can conclude:

$$\mathrm{E}\big[\frac{\hat{\alpha}_{Y*} - \hat{\theta}_{00}}{\hat{\theta}_{10}}\big] \approx \alpha_Y + \frac{1}{\theta_{10}^2}\Big[\alpha_Y \mathrm{Var}\big(\hat{\theta}_{10}\big) + \mathrm{Cov}\big(\hat{\theta}_{00}, \hat{\theta}_{10}\big)\Big].$$

Congruently, an approximation of the expected value of the estimator $\hat{\beta}_Y$ is given by:

$$\begin{aligned}\mathrm{E}\big[\frac{\hat{\beta}_{Y*} + \hat{\alpha}_{Y*} - \hat{\theta}_{01}}{\hat{\theta}_{11}} - \hat{\alpha}_Y\big] \approx \beta_Y &+ \frac{1}{\theta_{11}^2}\Big[(\beta_Y + \alpha_Y)\mathrm{Var}\big(\hat{\theta}_{11}\big) + \mathrm{Cov}\big(\hat{\theta}_{01}, \hat{\theta}_{11}\big)\Big] \\ &- \frac{1}{\theta_{10}^2}\Big[\alpha_Y \mathrm{Var}\big(\hat{\theta}_{10}\big) + \mathrm{Cov}\big(\hat{\theta}_{00}, \hat{\theta}_{10}\big)\Big].\end{aligned}$$

And the variance of the estimators is approximated by:

$$\begin{aligned}\mathrm{Var}\big(\frac{\hat{\alpha}_{Y*} - \hat{\theta}_{00}}{\hat{\theta}_{10}}\big) &\approx \frac{1}{\theta_{10}^2}\Big[\mathrm{Var}\big(\hat{\alpha}_{Y*}\big) + \alpha_Y^2 \mathrm{Var}\big(\hat{\theta}_{10}\big) + \mathrm{Var}\big(\hat{\theta}_{00}\big) + 2\alpha_Y \mathrm{Cov}\big(\hat{\theta}_{00}, \hat{\theta}_{10}\big)\Big], \\ \mathrm{Var}\big(\frac{\hat{\beta}_{Y*} + \hat{\alpha}_{Y*} - \hat{\theta}_{01}}{\hat{\theta}_{11}} - \hat{\alpha}_Y\big) &\approx \frac{1}{\theta_{11}^2}\Big[\big(\beta_Y + \alpha_Y\big)^2 \mathrm{Var}\big(\hat{\theta}_{11}\big) + \mathrm{Var}\big(\hat{\beta}_{Y*}\big) + \mathrm{Var}\big(\hat{\alpha}_{Y*}\big) \\ &+ 2\mathrm{Cov}\big(\hat{\alpha}_{Y*}, \hat{\beta}_{Y*}\big) + \mathrm{Var}\big(\hat{\theta}_{01}\big) + 2\big(\beta_Y + \alpha_Y\big)\mathrm{Cov}\big(\hat{\theta}_{11}, \hat{\theta}_{01}\big)\Big] \\ &+ \mathrm{Var}\big(\hat{\alpha}_Y\big).\end{aligned}$$

Note that in the case of differential measurement error, we assume that $\mathrm{Cov}\big(\hat{\theta}_{11}, \hat{\theta}_{00}\big) = 0$, $\mathrm{Cov}\big(\hat{\theta}_{11}, \hat{\theta}_{10}\big) = 0$, $\mathrm{Cov}\big(\hat{\theta}_{01}, \hat{\theta}_{00}\big) = 0$ and $\mathrm{Cov}\big(\hat{\theta}_{01}, \hat{\theta}_{10}\big) = 0$.

References

[1] Poehlman ET, WF Denino, T Beckett, et al. Effects of endurance and resistance training on total daily energy expenditure in young women: a controlled randomized trial. *J Clin Endocrinol Metab.* 2002;87(3):1004–1009.

[2] Plasqui G, Westerterp KR. Physical activity assessment with accelerometers: an evaluation against doubly labeled water. *Obesity.* 2007;15(10):2371–2379.

[3] Bijlsma JWJ, Welsing PMJ, Woodworth TG, et al. Early rheumatoid arthritis treated with tocilizumab, methotrexate, or their combination (U-Act-Early): a multicentre, randomised, double-blind, double-dummy, strategy trial. *Lancet.* 2016;388(10042):343–355.

[4] Prevoo ML, van 't Hof MA, Kuper HH, van Leeuwen MA, van de Putte LB, van Riel PL. Modified disease activity scores that include twenty-eight-joint counts development and validation in a prospective longitudinal study of patients with rheumatoid arthritis. *Arthritis Rheum.* 1995;38(1):44–48.

[5] Pincus T, Yazici Y, Sokka T. Complexities in assessment of rheumatoid arthritis: absence of a single gold standard measure. *Rheum Dis Clin North Am.* 2009;35(4):687–697.

[6] Anderson J, Caplan L, Yazdany J, et al. Rheumatoid arthritis disease activity measures: American College of Rheumatology recommendations for use in clinical practice *Arthritis Care Res.* 2012;64(5):640–647.

[7] Choy EH, Khoshaba B, Cooper D, Macgregor A, Scott DL. Development and validation of a patient-based disease activity score in rheumatoid arthritis that can be used in clinical trials and routine practice. *Arthritis Rheum.* 2008;59(2):192–199.

[8] Makrides M, Crowther CA, Gibson RA, Gibson RS, Skeaff CM. Efficacy and tolerability of low-dose iron supplements during pregnancy: a randomized controlled trial. *Am J Clin Nutr.* 2003;78(1):145–153.

[9] Davidson R, MacKinnon JG. *Econometric Theory and Methods.* New York, NY: Oxford University Press; 2004.

[10] Carroll RJ, Ruppert D, Stefanski LA, Crainiceanu CM. *Measurement Error in Nonlinear Models: A Modern Perspective.* 2nd ed. Boca Raton, FL: Chapman & Hall/CRC; 2006.

[11] Buonaccorsi JP. Measurement errors, linear calibration and inferences for means. *Comput Stat Data Anal.* 1991;11(3):239–257.

[12] Long JS, Ervin LH. Using heteroscedasticity consistent standard errors in the linear regression model. *Am Stat.* 2000;54(3):217–224.

[13] Buonaccorsi JP. *Measurement error: Models, Methods, and Applications.* Boca Raton, FL: Chapman & Hall/CRC; 2010.

[14] Franz VH. Ratios: A short guide to confidence limits and proper use. arXiv:0710.2024.

[15] Efron B. Bootstrap methods: another look at the jackknife. *Ann Statist.* 1979;7(1):1–26.

[16] Fieller EC. The biological standardization of insulin. *J Roy Stat Soc Suppl.* 1940;7(1):1–64.
